# What information is used in treatment decision aids? A systematic review of the types of evidence populating health decision aids

**DOI:** 10.1186/s12911-017-0415-7

**Published:** 2017-02-23

**Authors:** Amanda M. Clifford, Jean Ryan, Cathal Walsh, Arlene McCurtin

**Affiliations:** 1 Department of Clinical Therapies, Health Sciences Building, University of Limerick, Limerick, Ireland; 2 Department of Mathematics and Statistics, Health Research Institute, University of Limerick, Limerick, Ireland

**Keywords:** Evidence based practice, Decision aids, Shared decision making, Research evidence, Practice evidence, Patient evidence, Contextual factors

## Abstract

**Background:**

Patient decision aids (DAs) are support tools designed to provide patients with relevant information to help them make informed decisions about their healthcare. While DAs can be effective in improving patient knowledge and decision quality, it is unknown what types of information and evidence are used to populate such decision tools.

**Methods:**

Systematic methods were used to identify and appraise the relevant literature and patient DAs published between 2006 and 2015. Six databases (Academic Search Complete, AMED, CINAHL, Biomedical Reference Collection, General Sciences and MEDLINE) and reference list searching were used. Articles evaluating the effectiveness of the DAs were appraised using the Cochrane Risk of Bias tool. The content, quality and sources of evidence in the decision aids were evaluated using the IPDASi-SF and a novel classification system. Findings were synthesised and a narrative analysis was performed on the results.

**Results:**

Thirteen studies representing ten DAs met the inclusion criteria. The IPDASI-SF score ranged from 9 to 16 indicating many of the studies met the majority of quality criteria. Sources of evidence were described but reports were sometimes generic or missing important information. The majority of DAs incorporated high quality research evidence including systematic reviews and meta-analyses. Patient and practice evidence was less commonly employed, with only a third of included DAs using these to populate decision aid content. The quality of practice and patient evidence ranged from high to low. Contextual factors were addressed across all DAs to varying degrees and covered a range of factors.

**Conclusions:**

This is an initial study examining the information and evidence used to populate DAs. While research evidence and contextual factors are well represented in included DAs, consideration should be given to incorporating high quality information representing all four pillars of evidence based practice when developing DAs. Further, patient and expert practice evidence should be acquired rigorously and DAs should report the means by which such evidence is obtained with citations clearly provided.

**Electronic supplementary material:**

The online version of this article (doi:10.1186/s12911-017-0415-7) contains supplementary material, which is available to authorized users.

## Background

Patients are often required to make important decisions about their treatment that will have a direct impact their health [1, 2]. The provision of relevant information about available treatment options and likely outcomes empowers patients to make fully informed decisions and determine their preferred options [3]. Shared decision making is an approach where patients make decisions together with their healthcare practitioner using the best available evidence [4]. This approach promotes patient engagement in the decision making process, enables patients communicate their preferences and chose the best treatment option having considered different alternatives [4–6]. A means of facilitating shared decision making is through the use of decision support tools. However, some decisions can be complex due to a lack of evidence on treatment effectiveness and difficulties finding the balance between the benefits and harms of respective treatment options [3, 7]. Thus, a major challenge to shared decision making is ensuring decision support materials are comprehensive and provide relevant information that represents the totality of best available evidence. Decision aids (DAs) are decision support tools ‘*designed to help patients make decisions by providing information on the options and outcomes relevant to a person’s health status*’ [5]. Effectively, a DA brings together different types of knowledge about the intervention being offered in order to assist the patient to make fully informed decisions regarding that treatment. DAs contribute to patients being actively involved in their care and have been shown to lead to higher decision quality, increased patient knowledge and improved congruency between patient values and the treatment chosen [3, 5, 6]. Further the inclusion of DAs in consultations make them superior than those that depend on the spoken word alone, which have the potential to confuse patients [8]. Despite the effectiveness of DAs, there are concerns about the type and level of evidence being used to populate these decision tools and inconsistencies in evidence sources reported [9, 10].

A recent examination by Montori et al. [9] of 257 DAs found that even in the era of evidence based practice (EBP), approximately half provided citations for the research evidence used to inform their content [9]. These findings compare well however to an earlier review by Feldman-Stewart et al. [10] which identified only one fifth of DAs as providing a list of citations [10]. This suggests improvements in citation use over the intervening period by DA developers and may reflect improved methods and guidelines for DA development [11, 12]. Lack of transparent citing can lead to difficulties in deciphering the quality, accuracy and reliability of the information contained in DAs. Further, even when evidence sources are reported, there are indications of variations in the quality and quantity of evidence used as shown by Montori et al. [9]. This study reviewed a random sample of DAs (*n* = 20), identifying that while half used high quality evidence (systematic reviews, meta-analyses, numerous original research articles, clinical practice guidelines), others used less robust evidence (a narrative review, a singular piece of original research, expert opinion) [9] demonstrating a lack of standardisation in the evidence contained in DAs.

While research evidence is essential to guide decision making, it can be argued that it does not reflect the totality of evidence and is by itself not sufficient to facilitate patient-centred decision making [13]. EBP is a key contemporary model informing clinical practice and in its entirety can be proposed to comprise four pillars of evidence: research evidence, practice evidence, patient evidence and what can be termed contextual factors [14, 15]. Contextual factors reflect on pragmatic considerations such as cost, availability, policies and treatment burden and are considered highly important in influencing practice change [16]. Thus, it is purported that DAs should include information representing all four components to provide a more complete evidence based account of each treatment. This however, is not always the case. Feldman-Stewart [10] for example, identified that only 40% of DAs contained patient experiences [10] with many of these reflecting singular or a small number of patients’ accounts rather than summated high quality patient evidence.

It is suggested that use of evidence from the four components of EBP and the synthesis of such information in DAs is required in order to effectively contribute to fully informed patients and evidence based shared decision making. To the authors’ knowledge no published study has evaluated DAs using a comprehensive EBP framework. Thus, the aim of this review is to examine the evidence being used in DAs so as to fully understand what information patients receive to inform their treatment decisions when using DAs. For this purpose, the components of evidence based practice (EBP) including research evidence, practice evidence, patient evidence and contextual factors will be used to guide the evaluation. In addition, the quality and source of this information will also be established using a novel grading classification framework.

## Methods

This systematic review was conducted according to the PRISMA 2009 checklist (Additional file 1) [17]. Treatment DAs evaluated using RCTs were reviewed to examine if such decision tools reflect multiple forms of evidence.

### Literature Search and article inclusion

The literature search was conducted in November 2015. Search terms related to choice behaviour and decision support interventions were used to identify articles (see Table 1).

**Table 1:** Search Terms

1.	choice behav*/
2.	decision making/
3.	shared decision making/
4.	information seeking behav*/
5.	help seeking behav*
6.	or/1–5
7.	((decision) adj (support* or aid or tool or instrument or technolog* or technique* or system* or program* or algorithm* or process* or method* or intervention* or material* or board* or guide* or counselling*)).tw.
8.	((decision support) adj (system* clinical or technolog*)).tw.
9.	education technology/
10.	communication package/
11.	decision tree*/
12	((risk) adj (communication or assessment)).tw.
13.	((risk information) adj (tool or method)).tw.
14	((interactive) adj (health communication or booklet or graphic or tool)).tw.
15.	((informed) adj (choice or decision)).tw.
16.	or/7–15
17.	clinical trial/
18.	((randomized or randomised) adj (controlled trial)).tw.
19.	controlled clinical trial/
20.	randomized/
21.	randomised/
22.	placebo/
23.	random/
24.	trial/
25.	double blind method/
26.	or/17–25
27.	6 and 16 and 26

**Fig. 1:**
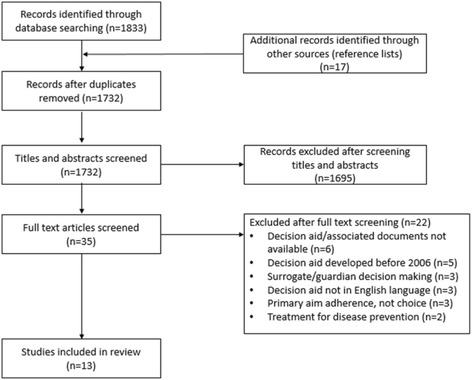
Prisma Diagram

The following six databases were searched using the above terms: Academic Search Complete, AMED, CINAHL, Biomedical Reference Collection, General Sciences and MEDLINE and reference list screening was also conducted to source further eligible articles (Fig. 1) including of large DA reviews such as Stacy et al. [5]. Search years were restricted to 2006–2015 in response to the publication of standards for decision aid development and evaluation [12]. The primary inclusion criteria were studies using RCTs to evaluate DAs and the availability of those DAs for content examination at a later stage (Table 2). Titles and abstracts were initially screened by one reviewer against predefined inclusion and exclusion criteria (Table 2) with articles overtly inapplicable to the review topic excluded. The remaining articles were independently (in isolation) double screened and categorised by each reviewer using a traffic light system of green (included), orange (potentially eligible), or red (excluded). The reviewers then met to agree final inclusions with disagreements being resolved through discussion with a third reviewer.

**Table 2:** Inclusion and exclusion criteria

Inclusion Criteria
• Studies investigating treatment choices for an established health condition
• Studies (randomised controlled trial design) comparing DAs to usual care, no intervention, alternative interventions
• Studies for which the decision aids being investigated were available to the research team for subsequent content examination
• Studies for which there was availability of associated documentation detailing development process of the decision aid
• Articles published in English language
Exclusion criteria
• Studies including hypothetical choices
• Decisions aids regarding:
○ clinical trial entry
○ screening/assessment
○ advance health care directives (e.g. resuscitation status)
○ educational programmes not directed toward decision making
○ promoting adherence only
○ eliciting informed consent only
• Decisions made by a surrogate or guardian for a patient
• Decision aids developed before 2006

### Data extraction and analysis

#### Bias

The Cochrane Risk of Bias tool was used to evaluate the articles related to the DAs across six domains: generation of random allocation sequence, concealment of allocation sequence, blinding, incomplete outcome data, selective outcome reporting and other biases [18]. Articles were assessed for bias by one reviewer with any ambiguity resolved through discussion with a second reviewer.

#### Quality of Decision aids

The short form of the International Patient Decision Aid Standards Instrument (IPDASi-SF) was used to assess the quality of the DAs [19, 20]. The IPDASi-SF comprises 16 items across seven dimensions, including: information, probabilities, values, development, disclosure, decision support technologies evaluation, and evidence. DAs were assessed by one reviewer with a second reviewer evaluating a random sample of 30% of DAs. Disagreements were resolved through discussion between the two reviewers.

#### Evidence used in DAs

As no tool existed to complete this task, a novel classification tool (Additional file 2) was developed by the authors to analyse the various types of evidence obtained through data extraction. The purpose of this tool was to determine the extent to which research, practice, patient and contextual evidence were incorporated within DA content, the methodological rigour used to obtain this evidence and the clarity of evidence sources within the DA. Supporting documentation (articles) was used to clarify/establish information where necessary. The extraction tool consisted of a hierarchy of classification criteria for each form of evidence. The criteria for research evidence was categorised according to an eight-point rubric based on evidence level and transparency ranging from A1 (systematic reviews of randomised clinical trials (RCTs), meta-analyses or multiple RCTs with sources available in DA or associated documents) to D2 (use of research evidence not evident). The criteria were based on the levels of evidence from the European Society of Cardiology guidelines [21] and aligned to the hierarchy of evidence outlined in the OCEBM Levels of Evidence tables [22]. Practice and patient evidence consisted of a seven-point rubric ranging from A1 (expert or clinical consensus of ≥15 participants obtained directly or through published evidence with evidence sources available in DA or associated documents) to D (no evidence included). Factors considered in classification related to the number of participants, the strength of methodology employed in obtaining the information and the clarity of evidence sources within the DA. Direction for a representative sample size was guided by recommendations for powering Delphi consensus, which suggests a minimum of 15 respondents [23]. Thus, the grading system awarded a higher score for studies including 15 or more participants for practice or patient evidence. Criteria for contextual factors were simplified to a three-point scale based on quantity ranging from A (includes multiple contextual factors e.g. time, cost, resources for each treatment option) to C (does not include any contextual factors). The tool was tested by three reviewers and went through an iterative process (five iterations) until the authors were satisfied that the criteria accurately represented the types of evidence and specific factors to be addressed. Data extraction and grading was completed by three reviewers independent from each other. One reviewer conducted extraction and grading for all DAs while two other reviewers evaluated a 50% sample each. Disagreements were discussed and resolved between the reviewing team.

## Results

### Results of search

The literature search yielded a total of 1732 unique citations with an additional 17 identified through reference list screening. Thirteen studies representing ten treatment DAs were included in the review [24–36]. Figure 1 depicts the flow of studies through the search process.

### Study characteristics

Eleven studies were conducted in North America [24–29, 31–34, 36], one in the UK [30] and one in Canada and Australia [35] thus predominately representing Anglo-Saxon countries. The DAs covered a range of health conditions including: cardiovascular disease [25, 29, 36], type 2 diabetes [25, 30, 33], osteoporosis [27, 31], breast cancer [26], cystic fibrosis [35], depression [28], post-traumatic stress disorder [32], uterine fibroids [34] and weight loss surgery in obesity [24]. DA interventions were delivered in a variety of formats including single page documents [25, 27, 29, 31, 36], booklets [30, 32, 35], cards [25, 28, 33], booklet and video [24, 34] and computer-based DAs [26]. Patients participating in the trials were from a range of settings including: primary care [25, 27–29, 31, 33], specialist clinics [26, 32, 34, 36], general practice [30], health plan systems [24] and outpatient centres [35] (Table 3).

**Table 3:** Study characteristics and main findings

General characteristics						Control arm	Intervention arm	Results
Publication	Context	Setting	Study design	Total no. of participants	Mean age	Control intervention	DA intervention	Main findings
Arterburn 2011 [21]	People considering bariatric surgery	Group Health Cooperative in USA	Prospective, randomised controlled trial	101	50.5	Booklet with general information on severe obesity and surgical weight loss treatments	Video and booklet about bariatric surgery + guidance (list of questions to ask clinician)	Both groups improved significantly in knowledge (*P* < 0.001), values concordance (*P* = 0.009), decisional conflict (*P* < 0.001) and decisional self-efficacy (*P* < 0.001). DA group had larger improvements than control group in knowledge (*P* = 0.03), decision conflict (*P* = 0.03), and outcome expectancies (*P* = 0.001). No difference in proportion of participants choosing bariatric surgery between groups.
Branda 2013 [22]	People with type 2 diabetes considering changing their antihyperglycaemic drugs or lipid lowering strategies	Rural primary care practices in USA	Cluster randomised controlled trial	103	57.6	Lipid therapy medication discussion OR Anti hyperglycaemic medication discussion	Diabetes Medication DA: decision cards with treatment information and usual care for lipid therapy OR Statin PtDA: 1-page DA with cardiovascular risk with and without medication, treatment information	DA group were more likely to report discussing medications (*p* < 0.001), answer knowledge questions correctly (risk reduction with statins, *p* = 0.07; knowledge about options *p* = 0.002) and were more engaged by their clinician in decision making (*p* = .01). No difference in patient satisfaction, decisional conflict, medication starts, adherence or clinical outcome
Jibaja-Weiss 2011 [23]	Women diagnosed with breast cancer considering surgical treatment	Two breast pathology clinics in USA	Randomised controlled trial	100	51	Breast cancer educational materials	Computer based information, values clarification and guidance (step by step process for making the decision)	DA group more likely to choose mastectomy (*P* = 0.018). No difference in satisfaction between groups. Decreased decisional conflict for both groups (*P* < 0.001) across assessment periods but DA group more informed about options (*P* = 0.007) and clearer about values (trend at *P* = 0.053) at pre-surgery assessment.
LeBlanc 2015 [24]	Women with osteopenia or osteoporosis	Primary care practices in USA	Randomised controlled trial	77	67.5	Clinicians discussed risk of fractures and treatment as usual without any research-related intervention OR clinicians were provided with patients’ individualised 10-year risk of bone fracture for use during the clinical encounter	1-page decision aid with personalised risk of having a fracture with and without medication and information about harms and side effects	DA group had better knowledge (*P* = 0.01), improved understanding of fracture risk and risk reduction with medication (*P* = 0.01 and *P* < 0.0001, respectively), increased patient involvement (*P* = 0.001) but had no effect on decisional conflict. Consultations using DA were 0.8 min longer. DA arm had more patients receiving and filling prescriptions (*P* = 0.07), medication adherence was no different across arms at 6 months.
LeBlanc 2015 [25]	People with moderate or severe depression	Primary care practices in USA	Cluster randomised trial	297	43.5	No access to decision aid	Seven laminated cards with information about treatments	DA significantly improved patients’ decision comfort (*P* = 0.02), knowledge (*P* = 0.03), satisfaction (*P* = 0.81 to *P* = 0.002, depending on domain) and involvement (*P* < 0.001) and clinicians’ decisional comfort (*P* < 0.001) and satisfaction (*P* = 0.02). No differences in consultation duration, adherence or improvement in depression control between groups.
Mann 2010 [26]	People diagnosed with diabetes considering statins to reduce cardiovascular risk	Primary care practices in USA	Cluster randomised trial	150	58	Pamphlet about reducing cholesterol through diet	1-page DA with cardiovascular risk with and without medication and information about statins	DA group more likely to accurately estimate risk of cardiac arrest without statin (OR: 1.9, CI: 1.0–3.8) and with statin (OR: 1.4, CI: 0.7–2.8). DA group reported stronger belief in the need for statins (OR 1.45, CI: 0.89–2.36) and were less likely to have concerns about long-term effects (OR: 0.44, CI: 0.20–0.97). DA resulted in improvements in decisional conflict (*P* = 0.1). No difference in statin adherence or knowledge between groups.
Mathers 2012 [27]	People with type 2 diabetes considering insulin therapy	General practices in UK	Cluster randomised controlled trial	175	64	No access to decision aid	Booklet containing information on treatment options, values clarification + guidance	DA group had lower total Decisional Conflict Scores (*p* < 0.001); greater knowledge (*p* < 0.001); realistic expectations (*p* < 0.001); and more autonomy in decision making (*p* = 0.012). No significant difference in the glycaemic control between groups.
Montori 2011 [28]	Postmenopausal women at risk of osteoporotic fractures	General medicine and primary care practises in USA	Multicentre, randomised controlled trial.	100	67	Review of bone mineral density results without fracture risk calculation or graphic representation of treatment benefit + general information booklet	1-page decision aid with personalised risk of having a fracture with and without medication and information about harms and side effects	DA group were 1.8 times more likely to correctly identify fracture risk, 2.7 times more likely to identify risk reduction with bisphosphonates and demonstrated improved involvement in decision making process by 23%. Bisphosphonates started more in DA group, adherence similarly high at 6 months, across both groups but proportion with more than 80% adherence was higher in DA group.
Mott 2014 [29]	War veterans with PTSD	PTSD clinic in USA	Randomised controlled trial	27	29.3	No access to decision aid	Booklet describing treatment options	Greater number of people in DA group preferred an evidence-based treatment and received an adequate dose of therapy compared to control (≥9 sessions). No difference in initiation rates of psychotherapy between groups.
Mullan 2009 [30]	People with type 2 diabetes considering treatment options	Primary care and family medicine sites in USA	Cluster randomised trial	85	62.1	12-page pamphlet on oral antihyperglycaemic medications	Six decision cards with information about treatments	DA group had better knowledge and more involvement in decision making. Similar scores for trust in physician and decisional conflict between groups. At follow-up, both groups had almost perfect medication use but there was no significant impact on HbA1c levels.
Solberg 2010 [31]	Women considering treatment options for uterine fibroids	Gynaecology clinics in USA	Randomised controlled trial	300	46	Pamphlet about uterine fibroids	DVD and booklet, decision worksheet and nurse coach access	DA group reported more options being mentioned, had better knowledge scores, were more likely to report being adequately informed and decisions were both more satisfactory and consistent with personal values.
Vandemheen 2009 [32]	People with cystic fibrosis considering lung transplantation	Outpatient centres in Canada and Australia	Single-blind, randomised controlled trial	149	30.4	Blank pages and a letter explaining why blank pages were included	Booklet with treatment information	DA group had better knowledge about options (*P* < 0.0001) and more realistic expectations (*P* < 0.0001). Decisional conflict was significantly lower in DA (*P* = 0.0007).
Weymiller 2007 [33]	People with type 2 diabetes	Metabolic clinic in USA	Cluster randomised trial	97	65	Standard educational pamphlet on cholesterol management	1-page DA with cardiovascular risk with and without medication and information about statins	DA group had better knowledge, estimated cardiovascular risk and potential absolute risk reduction with statin drugs, and had less decisional conflict. DA missed less doses than control group at 3-month follow-up.

### Cochrane Risk of Bias

A summary of the risk of bias is shown in Table 4. Sequence generation was deemed to be of low risk of bias in 8/13 (61.5%) studies [24, 26, 27, 31–33, 35, 36]. Selective reporting was deemed to be low risk in 8/13 studies as trial registration or protocols were available publicly [25, 27–31, 33, 35, 36] and unclear in the remaining. Incomplete outcome data was adequately described in 8/13 studies [27–31, 34–36]. Nine (69.2%) studies were deemed to be free of other sources of bias [24, 26, 27, 31–36], it being not possible to judge the remaining studies. Allocation concealment was rated as unclear in 9/13 studies [24–29, 32, 34, 36]. Blinding of participants and study personnel was unclear 11/13 (84.6%) studies [24–27, 29–35]. Four individual studies reported on bias as follows: Branda [25] noted the possibility for selection bias due to incomplete recruitment of patients and clinicians within each cluster [25]; LeBlanc [28] reported a 20% loss to follow-up at primary end-point which may have increased risk of bias in favour of intervention [28]; Mann [29] stated that clustering effects were not adjusted but did not report on change in data as a result of this [29], and; Mathers [30] noted a potential recruitment bias as more participants were allocated to the intervention than the control group [30].

**Table 4:** Cochrane Risk of Bias results

Criteria	Sequence generation	Allocation concealment	Blinding (participants, personnel and outcome assessors)	Incomplete outcome data	Selective outcome reporting	Other sources of bias
Arterburn 2011 [24]	+	?	?	?	?	+
Branda 2013 [25]	?	?	?	?	+	?
Jibaja-Weiss 2011 [26]	+	?	?	?	?	+
LeBlanc 2015 [27]	+	?	?	+	+	+
LeBlanc 2015 [28]	?	?	-	+	+	?
Mann 2010 [29]	?	?	?	+	?	?
Mathers 2012 [30]	?	+	?	+	+	?
Montori 2011 [31]	+	+	?	+	+	+
Mott 2014 [32]	+	?	?	?	?	+
Mullan 2009 [33]	+	+	?	?	+	+
Solberg 2010 [34]	?	?	?	+	?	+
Vandemheen 2009 [35]	+	+	?	+	+	+
Weymiller 2007 [36]	+	?	+	+	+	+

+ Low risk; ? Unclear risk; - High risk

### Decision Aid Quality (IPDASi-SF)

The ten unique DAs were assessed using the IPDASi-SF. The rationale for scores is explained fully in previous research [19]. Overall, the DA scores ranged from 9 to 16 (mean 13 ± 2.11) thus ranging from moderate to excellent quality (Table 5). All DAs for example, met the criteria related to information with all aids scoring maximum points in this section. DAs also scored highly on items related to development including the assessment of patient needs (9/10) and completing testing with patients (7/10). DAs reported a positive effect on user knowledge (9/10) and decision quality (7/10). The reporting of event rates (the communication of the likelihood of an outcome occurring) was completed least often (5/10).

**Table 5:** IPDASi-SF results

Publication	Arterburn 2011 [21]	Jibaja-Weiss 2011 [23]	LeBlanc 2015 [24]Montori 2011 [28]	LeBlanc 2015 [24]	Mathers 2012 [27]	Mott 2014 [28]	Mullan 2009 [30]Branda 2013 [22]	Solberg 2010 [31]	Vandemheen 2009 [32]	Weymiller 2007 [33] Mann 2010 [26] Branda 2013 [22]
Production Year	2014 (update)	2006	2007	2012	2008	-	-	2014 (update)	2006	2007
Information										
Options available	+	+	+	+	+	+	+	+	+	+
Positive features	+	+	+	+	+	+	+	+	+	+
Negative features	+	+	+	+	+	+	+	+	+	+
Fair comparison	+	+	+	+	+	+	+	+	+	+
Probabilities										
Reference class	+	-	+	+	+	+	+	+	+	+
Event rates	+	-	+	-	+	-	-	-	+	+
Compare probabilities	+	-	+	+	+	+	-	+	-	+
Values										
Personal importance	+	+	-	-	+	+	-	+	+	-
Development										
Patients’ needs	+	+	+	+	+	-	+	+	+	+
Impartial review	+	+	+	+	+	+	+	+	+	+
Tested with patients	-	+	+	+	+	+	+	-	-	+
Disclosure										
Information about funding	+	+	+	+	+	+	+	+	+	+
DST evaluation										
Knowledge	+	+	+	+	+	-	+	+	+	+
Improved decision quality	+	+	-	+	+	-	-	+	+	+
Evidence										
Citations to studies	+	+	+	+	+	-	+	+	+	+
Production date	+	+	+	+	+	-	-	+	+	+
Total	15	13	14	14	16	10	11	14	14	15

+ Met criterion; ? Unclear; - Unmet criterion

### Evidence levels and sources in decision aids (novel framework)

Table 6 outlines the types of evidence retrieved from the included DAs.

**Table 6:** Evidence used in decision aids

Publications	Decision aid	Pillar of evidence			
		Research evidence	Practice evidence	Patient evidence	Contextual evidence
Arterburn 2011 [24]	Weight Loss Surgery: Is it right for you?	A1	B1	A1	B
Jibaja-Weiss 2011 [26]	A Patchwork of Life: One Woman’s Story	A1	D	B2	B
LeBlanc 2015 [27], Montori 2011 [31]	Osteoporosis Choice	A1	C2	D	B
LeBlanc 2015 [28]	Depression Medication Choice	A1	D	D	B
Mathers 2012 [30]	Starting Insulin. Your Choice.	A2	D	D	B
Mott 2014 [32]	Getting help for PTSD: A guide to finding the right treatment for you	D1	D	D	B
Mullan 2009 [33], Branda 2013 [25]	Diabetes Medication Choice	A1	D	D	B
Solberg 2010 [34]	Treatment choices for uterine fibroids	A1	B1	A1	B
Vandemheen 2009 [35]	When your lung function is getting worse… Should you be referred for a lung transplant? A decision aid for adults with cystic fibrosis	D1	D	D	A
Weymiller 2007 [36], Mann 2010 [29], Branda 2013 [25]	Statin Choice	A1	D	D	B

#### Research evidence

Research evidence was well represented in most DAs evaluated (Table 6). The majority (7/10) incorporated a range of research evidence including articles of high methodological rigour such as systematic reviews, meta-analyses or randomised controlled trials. One DA was categorised as an A2 as it used a combination of national clinical guidelines and a cohort study to populate content [30]. In some cases, it was unclear as to the type and rigour of research evidence used to inform DA content and this was reflected in categorisation. Mott et al. [32] for example, referred to research findings but did not provide citations to studies used [32]. Vandemheen (2010) reported that the DA content was based on a literature review, patient interviews and data from a national registry [35] but did not delineate the sources of all DA information.

#### Practice Evidence

Use of practice evidence to populate DA content was less evident being incorporated into only 3/10 DAs (Table 6). Two DAs, produced by the same organisation, were categorised as B1 on the grading rubric as they directly incorporated clinicians’ opinions through interview methods [24, 34]. Despite this, both DAs recruited less than 15 clinicians/experts and therefore did not receive the maximum score. One DA [27, 31] was graded as C2 despite incorporating practice evidence as it utilised an unknown number of members of the research team to retrieve this evidence which was also not presented explicitly within the aid and used mainly to supplement low level research evidence about the specific topic [37].

#### Patient evidence

A third (3/10) of DAs integrated patient evidence as a source of information (Table 6). Two DAs, produced by the same organisation scored A1 in this category interviewing 30 people considered to represent the target audience [24, 34]. The methodology, number of participants and explicit presentation of patient evidence justified the highest grade for this category. The other DA graded as B2 as it incorporated hypothetical patient stories played by actors but did not report on how the script was developed [26].

#### Contextual factors/evidence

Contextual factors were generally addressed to some degree by all DAs (Table 6). The majority (9/10) typically presented 1–2 contextual factors per treatment option. One DA presented more than two [35]. Issues addressed by DAs aids included: daily routine (6/10) including dose and particulars such as how to take medication, cost (5/10), length of hospital stay, if any (3/10), time to execute treatment including consultation length and/or number of weekly sessions involved (2/10), and other practical issues e.g. self-monitoring (1/10), waiting time for procedure (1/10) and extra testing or doctor visits (1/10) as a result of the treatment.

## Discussion

The DAs examined in this study reflect a highly specified rather than generic group i.e. they were treatment DAs (they did not for example represent those used for assessment), representing Anglo-Saxon countries, evaluated using RCTs which had supporting documentation available in the form of published papers and technical documents. Thus, the review focused on a small number of DAs which met these criteria and the study findings should be interpreted with this in mind.

### Quality and transparency of DAs

The purpose of the IPDASi-SF is to present an overall impression of quality based on whether DAs contain suggested components and complete a rigorous development and evaluation process. According to the IPDASi-SF, the DAs reviewed in this study were of generally good quality, with many aids scoring maximally on rigour of development. This may be partially explained given that the evaluation of research content according to this tool refers predominately to the provision of research citations and not for example, the appropriate use of research to inform DA content. Thus, the IPDASi-SF differs from the novel classification tool developed for this review which assessed all evidence types and determined the quality of evidence according to evidence type and transparency. This demonstrated that a high score on the IPDASi-SF did not necessarily correlate with a high classification grade on using the novel classification tool used in this review. This suggests that while the quality of DAs as assessed using the IPDASi-SF may be sound, the variety of evidence used and the rigour of evidence sources other than research evidence are not guaranteed.

The majority of DAs provided citations for research evidence used to populate content either in the DA itself or in the supporting documentation representing variety in reporting methods. Two DAs developed by the same organisation, provided references within the DA [24, 34]. For one DA, the author was contacted to retrieve this information as there was no source references provided [32]. The remaining six DAs reported the sources of evidence either in papers detailing the development of the DA or in unpublished technical or background documents. In some cases, it was not clear whether citations were provided for *all* sources of information used to inform content. For example, in one DA citations were only provided for one of three treatment options included [30] the resultant score reflecting this. Thus, it was frequently challenging to assign DAs a score due to the lack of transparency in reporting evidence sources. Such findings are in line with similar results from a review of health information for patients (which includes DAs), which identified a high proportion of health information failed to disclose their evidence sources [9, 10]. It can be considered more surprising in this case given that the DAs included in this study had supporting documentation available. For patient, practice and contextual evidence, while sources were often available in associated documentation, reporting of this information was often generic and sometimes confusing. Given the importance of making correct and appropriate treatment decisions, evidence sourcing in DAs should without doubt be made explicit and transparent in order to allow the various stakeholders interpret information and make judgements about its usefulness and reliability, thus enabling patients to make more informed decisions.

### Content of Decision Aids

#### Research evidence and contextual factors

This novel review found that the information used to inform the content of the ten DAs examined is primarily founded on research evidence and contextual factors. Research evidence was well represented across the DAs evaluated, the majority using high quality research evidence categorised according to the A descriptor. This compares to the findings of a systematic review by Montori et al. [9], which found that half of the DAs examined used high quality evidence [9]. Furthermore, for the DAs examined the research evidence was explicit as citations for research evidence were provided in almost all aids. This finding compares favourably to previous research, which identified between a fifth (21%) to half (50%) of DAs provided citations for research evidence [9, 10]. The difference in findings may be explained by a number of factors, specifically the particular nature (treatment) of the DAs included in each study, the relative recency of those in the current study which reflect on the application of the 2006 IPDAS standards [12] and the bias that may result from including only DAs which had supporting documentation. While this was necessary to examine DA content in depth, it also limited inclusion and therefore may not represent all available DAs, implying that Montori et al’s [9] and Feldman-Stewart et al’s [10] findings may be more representative of the general pool of DAs. Topics regarding the medical condition, treatment options, expected outcomes and side effects were generally informed by research evidence reflecting standards advocated by International Standards for Patient Decision Aids Collaboration [12]. DAs which scored lower in this domain did so either because of lack of availability of high quality evidence or by not providing sufficient information to enable an evaluation to be made of the quality and sources of evidence. One DA reported using treatment manuals and a national website dedicated to the condition for treatment information [32] raising issues of information bias and comprehensiveness. In order to facilitate fully informed decision making by patients, up-to-date and complete scientific evidence should be used in DA content [10] which can be achieved via a systematic process of reviewing the literature and explicitly reporting scientific uncertainty where applicable [1]. Notably, most of the randomised controlled trials evaluating the effectiveness of the DAs were mainly of unclear bias, demonstrating that even when the appropriate study design is implemented it does not guarantee the quality of evidence within the DA itself.

As with research evidence, contextual evidence was reported in all aids with a wide range of factors being addressed and each DA containing at least one such factor. The inclusion of contextual information, particularly those representing treatment burden such as out-of-pocket costs and treatment intensity, can be seen as highly patient-centric given that such factors have the potential to dominate patient decision making [38]. DAs which include such content are likely to be more patient relevant and user friendly. Furthermore, complementing research evidence with matters related to the specific sociocultural context of the patient may serve to improve patient engagement with information [39].

#### Practice evidence and patient evidence

Practice and patient evidence were conversely not well represented in DA content with only a small subset of DAs incorporating this type of evidence. Further, the quality of such evidence varied and the sourcing of this information was not always transparent suggesting less rigorous rules for obtaining and using such evidence. Practice evidence was least commonly used to populate DAs with the majority (70%) not incorporating any clinical or expert evidence. This absence exists despite findings which demonstrate that patients value the opinion of health professionals when making decisions [40] and often regard clinical opinion as more important than information on intervention risks and benefits [41]. This relative lack of inclusion of practice evidence in DAs may be due to a number of factors including the perception of clinical expertise as a less reliable or significant form of evidence [22, 42]. It may also emanate from a perception that asking clinicians to comment on the aid during the design phase (rather than contribute collectively to the content) constitutes practice evidence. Additionally, the patient-clinician conversation itself when using DAs may be interpreted as utilising practice evidence suggesting that, unlike with research evidence, a singular clinical opinion may be considered sufficient to represent this form of evidence. Of the DAs in this study that incorporated practice evidence, two used clinicians to present factual information, such as describing treatment features, as well as report their clinical opinions [24, 34]. There was a lack of transparency with regards to how such experts were selected. Another DA supplemented weak research evidence about medication side-effects with clinical knowledge from the design team. This was retrieved through discussion and it is not known how many clinicians were consulted in the process [37]. Thus, in both cases issues with methodology and rigour in attaining practice evidence were present. The differences observed in retrieval and use between practice and research evidence suggests different value systems for different types of evidence. Practice evidence when used appears not to be rigorously obtained through formal methodologies, rather being frequently obtained through members (often few in number and unspecified) of the DA design team. Moreover, it appears primarily used as a supplementary source of information (especially when research evidence is lacking) rather than in its own right. As for research evidence, it is no less important to understand how and where from practice evidence is retrieved in order to ensure unbiased and systematically obtained information is delivered to patients [1].

The use of patient evidence in DAs is a contentious area with lack of consensus regarding its optimal use [39, 43]. This is reflected in the limited use of patient evidence in the DAs examined with the majority, (70%) not incorporating patient experiences and opinions. This is analogous to findings regarding practice evidence suggesting a corresponding low valuing for this type of information. It is in contradiction to patient evidence having been shown to facilitate patient understanding, coping with illness and adjustment to treatment [44–46], it being a useful means of providing insight into a particular experience about a condition. Research suggests that patients experiencing a range of health conditions have expressed positive responses to inclusion of patient evidence in health information and would like it to be integrated in health education [44]. There are clear suggestions of the relative valuing of such information by patients: they regard narrative information to be as credible as research evidence, and; patient narratives are suggested to be more persuasive than research evidence with patients often basing their treatment decision on the experiences of others above factual representations [43, 47, 48]. Thus, decision aid developers may purposely limit the focus on patient evidence in DA content both because of lowly valuing of such information and due to concerns around its optimal use for promoting unbiased, informed decisions [43]. These concerns may be balanced by involving patients in DA development from the offset for example, by providing input on the range of outcomes that patients consider critical in decision making (in addition to researcher-driven outcomes) and incorporating user panels comprising patients and caregivers (in addition to clinicians, experts and researchers) in an iterative design process focused on representing and balancing all forms of evidence [13]. Inclusion of patient evidence in DAs would address the real life experiences of decision aid users [49], thus empowering patients to make decisions based on a range of information that considers their individual context, goals, values and preferences.

The reduced practice and patient content in DAs may further reflect under researching (and thus lack of availability) of these forms of evidence. Thus, there is a requirement for both forms of evidence to be acquired using established research methods. Such methodologically rigorous research would help improve the reliability and validity of such evidence in addition to strengthening the relevant content used in DAs. It would also be useful when evaluating DAs which have been developed with the totality of evidence in mind, that studies be undertaken to examine how patients’ decisions are influenced by such individual forms of evidence. Further, as Violette et al. [50] point out, the content of DAs needs to remain current [50] to reflect additional and evolving evidence and policy developments.

These recommendations reflect a global move toward patient inclusion in their health care in a variety of ways specifically regarding fully informed shared decision making. As for other decision support tools, the development of DAs is a positive step in this regard and their importance is highlighted in policy and legal developments such as The Patient Protection and Affordable Care Act (2010) in the United States of which Section 3506 calls for the creation of a certification and quality process for patient decision aids and for best practices which include the sharing of developed tools [51]. Such quality processes should include the inclusion of multiple forms of evidence, rigorously acquired evidence irrespective of type and explicit sourcing of information used in DA content. To further progress this constructive movement, it is important moving forward that information representativeness, transparency and selectivity in DAs are targeted in order to best and fairly meet patient needs.

### Limitations

As noted, DAs examined in this study reflect a targeted rather than generic group and inclusion for review was limited to those which were evaluated using RCTs which were published in the literature. Thus, the nature of the study meant a lot of DAs were not included in this review and the finding cannot be readily generalised to all DAs.

Due to the lack of available tools and need to assess the rigour of patient, practice and contextual evidence, a novel grading system was developed. While unvalidated, this tool went through five iterations before application and was both comprehensive and reflective of the issues being addressed. Further, assessment was completed blindly by two independent reviewers and results were cross-checked by a third reviewer in order to ensure rigor and robustness of methodology employed and results obtained.

This study primarily focused on representativeness of information content. Thus information accuracy such as appropriate use of citations was not included in the analysis at this stage.

## Conclusion

The results of the current review demonstrate that the content of treatment DAs is informed mainly by the results of high quality research evidence and some contextual factors with patient and practice evidence being rarely incorporated. The transparency of reporting evidence sources can also be problematic especially where multiple documents exist regarding the development of the aid. Where patient or practice evidence is used, there are seldom rigorous, research-based methods used for obtaining this information. The same rigour used to retrieve and select research evidence for DAs is required for other forms of evidence to reduce information bias, improve the quality of DAs and enhance informed decision making.

## Additional files

Additional file 1: Prisma 2009 Checklist. Reporting of study against PRISMA criteria. (DOC 63 kb)
Additional file 2: Levels of Evidence. Grading of different types of evidence for data analysis. (DOCX 15 kb)

## Abbreviations

DAs: Patient decision aids
EBP: Evidence based practice
IPDASi-SF: International patient decision aid standards instrument
PPI: Public and patient involvement
RCTs: Randomised clinical trials
