# Supplementary material for: What information is used in treatment decision aids? A systematic review of the types of evidence populating health decision aids
Source: BMC Med Inform Decis Mak. 2017 Feb 23;17:22. doi: 10.1186/s12911-017-0415-7 (PMC5322640; doi:10.1186/s12911-017-0415-7)
Supplement: Additional file 2: — Levels of Evidence. Grading of different types of evidence for data analysis. (DOCX 15 kb) [file 12911_2017_415_MOESM2_ESM.docx]

Additional file 2:

Levels of Evidence

| **Research evidence** | |
| --- | --- |
| Level A1  Level A2 | Systematic reviews of randomised clinical trials, meta-analyses or multiple randomised clinical trials with sources available in decision aid or associated documents |
|  | Systematic reviews of randomised clinical trials, meta-analyses or multiple randomised clinical trials but sources are not available in decision aid or associated documents |
| Level B1  Level B2 | Systematic reviews of non-randomised studies, a single randomised clinical trial or large non-randomised studies with sources available in decision aid or associated documents |
|  | Systematic reviews of non-randomised studies, a single randomised clinical trial or large non-randomised studies but sources are not available in decision aid or associated documents |
| Level C1  Level C2 | Small non-randomised studies, retrospective studies or registries with sources available in decision aid or associated documents |
|  | Small non-randomised studies, retrospective studies or registries but sources are not available in decision aid or associated documents |
| Level D1  Level D2 | Research evidence elicited but sources unclear |
|  | Use of research evidence not evident |

| **Practice evidence** | |
| --- | --- |
| Level A1  Level A2 | Expert or clinical consensus (≥15 participants) obtained directly or through published evidence with evidence type explicitly presented and evidence sources are available in decision aid or associated documents |
|  | Expert or clinical opinion (≥15 participants) obtained directly or through published evidence but evidence type is not explicitly presented and/or evidence sources are not available in decision aid or associated documentation |
| Level B1  Level B2 | Expert or clinical opinion (<15 participants) obtained directly or through published evidence with evidence type explicitly presented and evidence sources are available in decision aid or associated documentation |
|  | Expert or clinical opinion (<15 participants) obtained directly or through published evidence but evidence type is not explicitly presented and/or evidence sources are not available in decision aid or associated documentation |
| Level C1  Level C2 | Expert or clinical opinion elicited through non-research based or informal methods with evidence type explicitly presented in decision aid and evidence sources are available in decision aid or associated documentation |
|  | Expert or clinical opinion elicited through non-research based or informal methods but evidence type is not explicitly presented and/or sources are not available in decision aid or associated documentation |
| Level D | No expert opinion |

| **Patient evidence** | |
| --- | --- |
| Level A1  Level A2 | Patient consensus (≥ 15 participants) obtained directly or through published evidence with evidence type explicitly presented and evidence sources are available in decision aid or associated documentation |
|  | Patient consensus (≥ 15 participants) obtained directly or through published evidence but evidence type is not explicitly presented and/or sources are not available in decision aid or associated documentation |
| Level B1  Level B2 | Patient opinion (<15 participants) obtained directly or through published evidence with evidence type explicitly presented and evidence sources are available in decision aid or associated documentation |
|  | Patient opinion (<15 participants) obtained directly or through published evidence but evidence type is not explicitly presented and/or sources are not available in decision aid or associated documentation |
| Level C1  Level C2 | Patient opinion elicited through non-research based or informal methods with evidence type explicitly presented and evidence sources are available in decision aid or associated documentation |
|  | Patient opinion elicited through non-research based or informal methods but evidence type is not explicitly presented and/or sources are not available in decision aid or associated documentation |
| Level D | No patient opinion |

| **Pragmatic/Contextual Evidence** | |
| --- | --- |
| Level A | Includes multiple (>2) pragmatic/contextual factors (e.g. time, cost, resources) for each treatment option |
| Level B | Includes at least 1-2 pragmatic/contextual factors (e.g. time, cost, resources) for each treatment option |
| Level C | Does not include any pragmatic/contextual factors |
